# Validating a non-invasive technique for monitoring physiological stress in the samango monkey

**DOI:** 10.4102/ojvr.v87i1.1720

**Published:** 2020-02-27

**Authors:** Juan Scheun, Adrian S.W. Tordiffe, Kirsten Wimberger, Andre Ganswindt

**Affiliations:** 1 National Zoological Garden, South African National Biodiversity Institute, Pretoria, South Africa; 2 Department of Zoology and Entomology, Endocrine Research Laboratory, Mammal Research Institute, Faculty of Natural and Agricultural Science, University of Pretoria, Pretoria, South Africa; 3 Department of Paraclinical Sciences, Faculty of Veterinary Science, University of Pretoria, Onderstepoort, Pretoria, South Africa; 4 The Cape Parrot Project, The Wild Bird Trust, Parktown, Johannesburg, South Africa

**Keywords:** ACTH challenge, animal welfare, samango monkey, non-invasive hormone monitoring, glucocorticoids, biological validation

## Abstract

The non-invasive monitoring of physiological stress can provide conservation and wildlife managers with an invaluable tool for assessing animal welfare and psychological health of captive and free-ranging populations. A significant decrease in free-ranging primate populations globally and an increase in captive-housed primates have led to a need to monitor the stress and general welfare of these animals. We examined the suitability of three enzyme immunoassays (EIAs) for monitoring stress-related physiological responses in the samango monkey, *Cercopithecus albogularis erythrarchus*. We conducted an adrenocorticotropic hormone (ACTH) challenge on a male and female at the National Zoological Garden, Pretoria, South Africa. Individual faecal samples were collected 8 days pre- and post-ACTH administration and subsequently analysed for faecal glucocorticoid metabolite (fGCM) concentrations. During the study, biological stressors occurred for both the male and female. Two of the three EIAs tested (11-oxoetiocholanolone I and II) were able to reliably monitor fGCM alterations throughout the study period in both sexes. The 11-oxoetiocholanolone I EIA, however, had the lowest mean deviation from the calculated baseline value and was thus chosen as the preferred assay. Both the physiological activation of the stress response and the biological response to a stressor could be monitored with the chosen assay. The successful establishment of a reliable, non-invasive method for monitoring adrenocortical activity in *C. albogularis erythrarchus* will now allow conservationists, scientific researchers and wildlife managers to evaluate the level of stress experienced, and general welfare, by animals in captivity as well as free-ranging populations.

## Introduction

Stress can be defined as a stimulus that may threaten, or appear to threaten, homeostasis (Selye 1936). Here, the perceived stressor triggers the so-called stress response, an adaptive mechanism aimed at restoring homeostasis of an individual. The initial step of this response includes the activation of the hypothalamic-pituitary-adrenal (HPA) axis, resulting in the production and secretion of glucocorticoids (GCs), such as cortisol and corticosterone, into the bloodstream (see Sapolsky, Romero & Munck 2000). The temporal increase in plasma GC concentrations is responsible for the necessary adjustment of individual metabolism, increasing energy availability, enhancing cardiovascular activity and behavioural alterations, in order to restore homeostasis (Reeder & Kramer 2005). Although these adaptation processes can be beneficial in the short term, a prolonged exposure to elevated GC concentrations may lead to numerous deleterious effects, most notably the suppression of the immune system, memory impairment and reproductive suppression (Konstantinos & Sheridan 2001; Mcewen et al. 2008; Sheriff et al. 2009; Suter & Schwartz 1985). Because of their key role in the course of the stress response, GCs are often used as a physiological marker for the level of stress perceived by an animal (Palme 2019; Sheriff et al. 2011).

Although blood offers a robust matrix for measuring GC concentrations, the challenges associated with blood collection in the form of animal capture and restraint, along with the increase in GC concentrations as a result of the stressful procedure (feedback effect), render this approach impractical in many captive and free-ranging settings (Heistermann 2010). Consequently, monitoring adrenocortical activity using faeces as a hormone matrix has become an accepted non-invasive method for assessing the stress response of an animal. Faecal samples are comparatively easy to collect with little to no disturbance to the animal and as a result, sampling is feedback-free (Kersey & Dehnhard 2014). Further, faecal hormone values are less affected by episodic fluctuations of hormone secretions, as circulating hormone levels within the bloodstream accumulate within the faeces over an extended period of time (Ganswindt et al. 2012; McEwen & Wingfield 2003). However, as hormone metabolism and excretion may differ distinctively between species and sex (Goymann 2012; Rettenbacher et al. 2004), assays for non-invasive hormone monitoring need to be carefully validated in terms of its applicability for the intended hormone matrix to ensure a reliable quantification of respective hormone metabolites (Touma & Palme 2005). In this regard, the activation of the HPA axis, as a key part of the validation process, can be performed through biological and physiological means. In terms of biological stressors, factors such as animal handling, restraint, transportation and injury have all been shown to activate the HPA axis (Bosson, Palme & Boonstra 2013; Dehnhard et al. 2001; Ganswindt et al. 2010; Goymann et al. 1999; Hämäläinen et al. 2014). However, individual variation, in terms of the neuroendocrine response to the presence of a stressor, can vary considerably and should be taken into account when using a biological stressor for assay validation (see Gott et al. 2018; Koolhaas et al. 2010). The physiological validation is conducted through the artificial activation of the HPA axis; this is achieved by injecting an individual with synthetic adrenocorticotropic hormone (ACTH) to increase GC production (ACTH challenge; Palme 2019).

Primates are the mammal order most threatened by extinction (Schipper et al. 2008), with hunting and deforestation presenting the most common threats (Rovero et al. 2012). The forest dwelling samango monkey (*Cercopithecus albogularis)* in South Africa is one such species threatened by anthropogenic activities (Linden et al. 2016; Skinner & Chimimba 2005; Wimberger, Nowak & Hill 2017). Changes in faecal glucocorticoid metabolite (fGCM) concentrations, according to environmental and biological stressors, can be used to provide insight into how this species has adapted to anthropogenic threats. Analyses of GC in a related species, *Cercopithecus mitis stuhlmanni*, showed that fGCM concentrations increased when individuals ate less preferred items (fallback foods) and decreased when they ate preferred items (e.g. insects, fruits, young leaves; Foerster, Cords & Monfort 2012). Furthermore, this change was more substantial in those female monkeys in the energetically demanding stages of late pregnancy and early lactation. Similarly, the validation of an appropriate enzyme immunoassay (EIA) for monitoring fGCM concentrations in the Barbary macaque (*Macaca sylvanus*) showed that individual fGCM concentrations increased during interactions with tourists (Maréchal et al. 2011). Thus, the validation of an appropriate EIA for monitoring alterations in fGCM concentrations can offer an ideal tool for assessing the physiological reaction of primates to anthropogenic activities and changes in their natural environment.

The aim of this study was to establish a suitable non-invasive tool for a reliable monitoring of fGCM concentrations, as a measure of stress, in *C. albogularis erythrarchus*. Such a technique would be an ideal tool for assessing animal welfare of captive and free-ranging populations.

## Material and methods

### Adrenocorticotropic hormone challenge test

An ACTH challenge was conducted on an adult male (6 years) and adult female (5 years) samango monkey (*C. albogularis erythrarchus*) housed at the National Zoological Garden (NZG), Pretoria, South Africa, from 17 October to 02 November 2012. Both individuals were housed in separate, adjoining cages (6.5 m × 7.0 m × 3.5 m) since 2009, which allowed for direct visual, olfactory and vocal communication. Each cage contained a sleeping room, suspended walkways, climbing poles, ‘monkey bars’, large trees as well as a number of resting platforms. The study was conducted outside of the defined reproductive period of the species, negating the possible effect that reproductive hormones, and the resulting behaviours, may have on the adrenal activity of the study animals. Both animals were fed a mixed vegetable and fruit diet, while water was available *ad libitum*. Regular veterinarian assessments confirmed that both individuals were healthy at the beginning of the study.

To determine individual baseline fGCM concentrations, both cages were checked for faeces regularly and fresh faecal samples were collected from both individuals for a period of 7 days. Collected material was homogenised and a 5 g – 10 g portion frozen at -20 °C. On day 8 of the experiment, both individuals were net-caught and hand-injected with zolazepam or tiletamine (3.5 mg/kg – 5 mg/kg body weight, Zoletil^®^, Virbac, South Africa), before transferring them to the Veterinary Hospital at the NZG as part of an annual health assessment. Once at the veterinary hospital, both individuals were intubated and maintained under anaesthesia on 1% – 2% isoflurane in oxygen. While under anaesthesia, individual SpO_2_, EtCO_2_, blood pressure, electrocardiography as well as heart and respiratory rate were monitored. A physical examination, to account for any abnormalities or injuries, was conducted on both individuals. During the examination, the NZG veterinary staff confirmed a tail fracture in the male, which was presumably sustained during the net-capture event. The fracture was treated with a splinted bandage by the veterinarian and the animal received oral carprofen (Rimadyl®, Zoetis, South Africa) at 2.5mg/kg once a day for 5 days for pain management. Carprofen is a pain suppressor which has no direct effect on adrenal activity. The NZG staff monitored the tail recovery throughout the entire healing period; the need to change the bandage did not arise during the study period. Throughout the intubation period, balanced intravenous fluids (Ringer’s lactate) were administered to each individual at a rate of 10 mL/kg/h. A blood sample (10 mL) was collected from the caudal saphenous vein to obtain serum cortisol values. Following this, each individual was injected with 10 IU (1.1 IU/kg – 1.5 IU/kg) synthetic ACTH (Synacthen^®^, Novartis, Australia) intramuscularly. Forty minutes after the Synacthen injection, a second blood sample was taken to capture the induced rise in serum cortisol concentrations. Both individuals were allowed to recover in a darkened cage for 3 hours before being released back into their respective individual enclosures.

The male was released into a cordoned off section of its cage to allow the NZG staff to monitor his response to the tail bandage. This cordoned off section limited the direct contact (visual, olfactory) between study animals for the rest of the study. Both the tail injury and the limited visual contact between individuals were regarded as possible biological stressors. All individual faecal droppings were collected for 48 h post-ACTH administration. Following this, cages were checked for faeces regularly (5–10 times a day) for a further 5 days. In all cases, material from the middle of the dropping was collected to avoid any cross-contamination that may have occurred through contact with urine or any other environmental contaminants.

### Faecal sample extraction and analysis

All faecal sample extractions and analyses were conducted at the Endocrine Research Laboratory, University of Pretoria, South Africa. Faecal samples were lyophilised, pulverised and sieved through a fine mesh to remove any fibrous or undigested material (Fieß, Heistermann & Hodges 1999). Subsequently, 0.050 g – 0.055 g of the faecal powder was extracted by vortexing for 15 minutes with 1.5 mL 80% ethanol. Following centrifugation for 10 min at 1500 × *g*, the supernatants were transferred into new microcentrifuge tubes and stored at -20 °C until analyses.

The resulting steroid extracts were measured for fGCM concentrations using three EIAs: (1) an 11-oxoetiocholanolone I (detecting 11, 17-dioxoandrostanes), (2) an 11--oxoetiocholanolone II (detecting fGCMs with a 5β-3α-ol-11-one structure) and (3) a cortisol EIA. Initial assay selection was based on available EIAs already used for monitoring fGCM alterations in other non-human primates (e.g. Hämäläinen et al. 2014; Heistermann, Palme & Ganswindt 2006) including the vervet monkey (*Chlorocebus pygerythrus*) (Young et al. 2017). All assays were performed on microtiter plates as described by Scheun et al. (2016). Details for the three EIAs are described by Palme and Möstl (1997) for 11-oxoetiocholanolone I and cortisol, as well as Möstl et al. (2002) for 11-oxoetiocholanolone II assay. Sensitivities for all three EIAs used were 0.6 ng/g dry weight. Parallelism tests were performed for all three EIAs, with difference in slope being < 5% for the 11-oxoetiocholanolone I and cortisol, and < 3% for the 11-oxoetiocholanolone IIEIA. Intra- and inter-assay coefficients of variation, determined by repeated measurements of high- and low-value quality controls, ranged between 1.9% – 11.7% (11-oxoetiocholanolone I), 6.1% – 11.7% (11-oxoetiocholanolone II) and 9.5% – 11.4% (cortisol), respectively.

### Serum analysis

The two blood samples were put on ice for 30–60 min until clotted and subsequently centrifuged at 1500 × *g* for 15 min. The serum was then transferred into polystyrene tubes and stored at -20 °C until analysis at the Veterinary Hormone Laboratory, Faculty of Veterinary Science, University of Pretoria.

Serum cortisol concentrations were determined using a Coat-A-Count© cortisol Radio-immunoassay (Siemens Medial Solutions Diagnostics, Tarrytown, New York, United States [US]). In brief, 25 *µ*L standards, controls and samples were transferred in duplicates into coated tubes, respectively. One millilitre 125 I cortisol solution was added, and the tubes incubated for 45 min at 37 °C. Subsequently, all tubes were thoroughly decanted, patted dry and counted for 1 min in a gamma counter (Wallac Wizzard2, Perkin Elmer) using MULTICALC software. Sensitivity of the assay was 5.5 nmol/L. Cross-reactivities of the antibody used are provided in the assay instruction manual. Concentrations are given in mmol/L.

### Data analysis

Individual baseline fGCM concentrations were determined for the respective data sets resulting from the three EIAs employed using an iterative process (Brown et al. 1994; Scheun et al. 2016). Here, the mean and standard deviation (SD) value for each individual EIA-specific data set were calculated. Subsequently, all data points higher than the mean + 1.5 SD were removed and the mean and SD recalculated. This process was repeated until no value exceeds the mean + 1.5 SD, thus yielding the individual baseline value. Periods of elevated fGCM concentrations were defined as the occurrence of two or more consecutive samples that exceed the calculated individual baseline level. To calculate the baseline stability of each EIA, the mean absolute deviation (MAD) from the calculated baseline value was determined. Here, the calculated baseline fGCM value was subtracted from all pre-injection fGCM values for each EIA-specific data set. The differences were noted as absolute values and the mean of the values was calculated, which subsequently gave the MAD value for each EIA. The MAD values were converted to a percentage deviation value (MAD/Baseline Value*100) to allow for the comparison between EIAs.

Gut passage time in similar sized primates has been reported as 20–36 h (Bahr et al. 2000; Rimbach et al. 2013; Young et al. 2017). Thus, endocrine response to the induced physiological stressor has been evaluated by focusing on alterations in fGCM concentrations up to 48 h post-induction. Similarly, the endocrine response to the presence of the biological stressors focused on the alteration in fGCM concentrations from 48 to 191.5 h (male) and 48 to 199 h (female) post-injection. To determine whether an EIA could successfully monitor changes in fGCM concentration we compared the (1) highest signal following both stressors and (2) the lowest MAD value of the tested assays.

### Ethical considerations

The study was performed with the approval of the National Zoological Gardens Ethics Committee (Reference P10/27).

## Results

### Adrenocorticotropic hormone challenge test

Serum GC concentrations increased considerably following the ACTH injection, with a 65.5% (male, pre-injection: 1.06 mmol/L, post-injection: 1.78 nmol/L) and 35.7% (female, pre-injection: 1.70 mmol/L, post-injection: 2.30 mmol/L) increase observed after 40 min.

In total, 23 faecal samples from the male (13 pre- and 10 post-injection) and 19 faecal samples from the female (10 pre- and 9 post-injection) were collected during the monitoring period. For the male, the 11-oxoetiocholanolone I (53% ± 21.65% SD) had the lowest percentage MAD value compared to the 11-oxoetiocholanolone II (71.14% ± 25.52% SD) and cortisol EIA (54.21% ± 39.41% SD). The cortisol EIA (0.09% ± 0.24% SD) had the lowest percentage MAD value in the female, followed by the 11-oxoetiocholanolone II (0.30% ± 0.41% SD) and 11-oxoetiocholanolone I (0.51% ± 0.91% SD).

**FIGURE 1:**
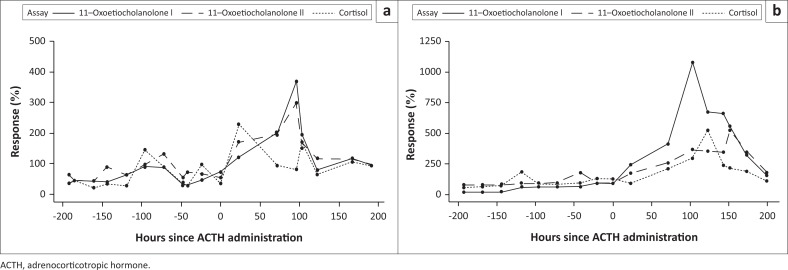
The longitudinal faecal glucocorticoid metabolite response for both male (a) and female (b) study animals following adrenocorticotropic hormone administration (time: 0) and separation or injury.

A considerable increase in fGCM concentrations occurred after 23 h post-injection for both study individuals (Figure 1). For the male, the most distinct increase in fGCM concentrations was detected using the cortisol EIA (129.2% increase), whereas the 11-oxoetiocholanolone I and II EIAs showed an increase of 21.3% and 71.1%, respectively (Table 1). For the female, the 11-oxoetiocholanolone I EIA showed the most distinct increase in fGCM concentrations (145.5%), followed by the 11-oxoetiocholanolone II EIA (75.5%), whereas the cortisol EIA showed a decrease in fGCM concentration (-5.0%) post-ACTH administration (Table 2).

**TABLE 1:** Male baseline faecal glucocorticoid metabolite concentrations, as well as the mean absolute deviation from baseline, for all three enzyme immunoassays employed during the study.

Male (EIA)	Baseline fGCM (fGCM concentration)	Physiological stressor ACTH injection		Biological stressor Injury and separation		
		Peak fGCM concentration (hours post-injection)	Peak response (%)	Peak fGCM concentration (hours post-injection)	Peak response (%)	Period of elevated fGCM concentrations (hours post-injection)
11,17 DOA	0.95	1.15 (23)	21.33	3.51 (96)	369.47	71.5–103
3a,11oxo-CM	1.16	1.98 (23)	71.09	3.46 (96)	198.28	71.5–191.5
cortisol	0.08	0.18 (23)	129.17	0.14 (103)	70.89	N/A

Note: The peak faecal glucocorticoid metabolite concentration, as well as the peak response (%) from the calculated baseline level, for the described physiological stressor, are shown. For the biological stressor, the peak faecal glucocorticoid metabolite concentration and time until the peak sample (post-injection) is shown. Additionally, the peak response, in per cent increase from baseline faecal glucocorticoid metabolite value, as well as the period of elevated faecal glucocorticoid metabolite levels during the biological stressor period, is indicated for each enzyme immunoassay. All faecal glucocorticoid metabolite values are in *µ*g/g dry weight.

11,17 DOA, 11-oxoetiocholanolone I; 3a,11oxo-CM, 11-oxoetiocholanolone II.

ACTH, adrenocorticotropic hormone; fCGM, faecal glucocorticoid metabolite; EIA, enzyme immunoassay.

**TABLE 2:** Female baseline faecal glucocorticoid metabolite concentrations, as well as the mean absolute deviation from baseline, for all three enzyme immunoassays employed during the study.

Female (EIA)	Baseline fGCM (fGCM concentration)	Physiological stressor *ACTH injection*		Biological stressor *Injury and separation*		
		Peak fGCM concentration (hours post-injection)	Peak response (%)	Peak fGCM concentration (hours post-injection)	Peak response (%)	Period of elevated fGCM concentrations (hours post-injection)
11,17 DOA	0.53	1.30 (23)	145.46	5.72 (103)	979.62	71.5–199
3a,11oxo-CM	1.78	3.12 (23)	75.47	9.31 (151)	423	71.5–199
cortisol	0.05	0.04 (23)	−5.04	0.24 (122.5)	424.85	71.5–173

Note: The peak faecal glucocorticoid metabolite concentration, as well as the peak response (%) from the calculated baseline level, for the described physiological stressor, are shown. For the biological stressor, the peak faecal glucocorticoid metabolite concentration and time until the peak sample (post-injection) is shown. Additionally, the peak response, in per cent increase from baseline faecal glucocorticoid metabolite value, as well as the period of elevated faecal glucocorticoid metabolite levels during the biological stressor period, is indicated for each enzyme immunoassay. All faecal glucocorticoid metabolite values are in *µ*g/g dry weight.

11,17 DOA, 11-oxoetiocholanolone I; 3a,11oxo-CM, 11-oxoetiocholanolone II.

ACTH, adrenocorticotropic hormone; fCGM, faecal glucocorticoid metabolite; EIA, enzyme immunoassay.

### Biological stressors

For the male, the tail fracture and separation event co-occurred with a considerable increase in fGCM levels shown by two of the three EIAs tested (Figure 1). The 11-oxoetiocholanolone I EIA demonstrated elevated fGCM concentrations 71.5–103 h post-injection, whereafter fGCM levels decreased to the calculated baseline value; a peak fGCM concentration (peak response: 269.46%) was found at 96 h post-injection (Table 1). Similarly, the 11-oxoetiocholanolone II EIA showed elevated fGCM concentrations 71–191.5 h post-injection, before returning to the calculated baseline fGCM levels. A peak fGCM concentration (peak response: 198.30%) was found at 96 h post-injection (Table 1). Although the cortisol EIA also showed a peak in fGCM concentrations at 103 h post-injection (peak response: 70.90%, Table 1), no two consecutive samples, between 48 and 191.5 h were found to be above fGCM baseline concentrations. As such, no period of elevated fGCM concentrations could be defined for this assay for the period the biological stressors occurred.

Following the new separation setting with limited visual, olfactory contact, the 11-oxoetiocholanolone I EIA showed the highest peak response in fGCM concentrations (979.62%) at 103 h post-injection for the female (Figure 1, Table 2). Similarly, both the 11-oxoetiocholanolone II and cortisol EIAs showed a peak response in fGCM concentration exceeding 400% of the respective calculated baseline fGCM values at 151 h and 122.5 h, respectively (Table 2). Determined fGCM concentrations did not return to baseline levels for any of the three EIAs during the post-injection observation period.

## Discussion

The ability of the three EIAs to detect a considerable increase in fGCM concentrations in response to the ACTH challenge, as well as the biological stressors monitored by default, confirms that monitoring alterations in fGCM concentrations can be used as a reliable measure of adrenal activity in *C. albogularis erythrarchus.*

To obtain baseline serum cortisol concentrations, blood collection should occur within 3 min of the original stressor (Martínez-Mota et al. 2008; Romero & Reed 2005). As a result of the prolonged handling process of the animals in this study, we did not collect our first blood sample within the suggested time period. As such, the first male and female blood sample collected prior to the ACTH administration of the study may not be indicative of baseline cortisol levels. Despite this, the comparison between the first and second blood samples can still be used as evidence that serum cortisol concentrations increase in *C. albogularis erythrarchus* following the stimulation of the adrenal cortex.

The peak in fGCM concentration, as a result of the ACTH administration, occurred 23 h post-injection for the male and female study animal. The 23 h time delay, from injection to excretion, corresponds with the gut passage time of similar sized primates, such as the Sykes’ monkey (*C. mitis albogularis*, 26.5 h, Foerster & Monfort 2010), the vervet monkey (*Chlorocebus pygerythrus*, 29 h, Young et al. 2017), the long-tailed macaque (*Macacafascicularis*, 22 h, Bahr et al. 2000) and brown spider monkeys (*Ateles hybridus*, 24 h, Rimbach et al. 2013). For the male, all three EIAs were indicative of an increase in HPA activity. However, only the 11-oxoetiocholanolone I and 11-oxoetiocholanolone II EIAs were able to monitor the induced increase in fGCM concentrations in the female, with the cortisol EIA displaying baseline fGCM values at 23 h post-injection.

The physical injury to the tail of the male individual during the net-capture event may well explain the prolonged elevation of fGCM concentrations post-injection for two of the three EIAs used. Here, the 11-oxoetiocholanolone I EIA displayed the highest percentage fGCM response, as well as a prolonged elevation in fGCM concentration, throughout the presumed biological stressor period. Similarly, the 11-oxoetiocholanolone II EIA also showed a considerable increase in fGCM concentrations, and the related period of elevated fGCM concentrations was even considerably longer than the period determined via the 11-oxoetiocholanolone I EIA. The decrease in fGCM concentration throughout the biological validation period across assays may well be as a result of the tail fracture healing, leading to the decrease in the severity of the stress response and GC production. Although numerous articles on the activation of the HPA axis in response to extrinsic and intrinsic factors, such as translocation, capture and handling, toxins and seasonality, exist (Baker, Gobush & Vynne 2013; Girard-Buttoz et al. 2009; Hämäläinen et al. 2014), only a few case studies describe the effect of physical injuries on adrenal activity. For example, Ganswindt et al. (2010) showed that physical injury in African elephants (*Loxodonta africana*) increased fGCM concentrations, which returned to baseline levels following recovery. Likewise, Kumar et al. (2014) found that Asian elephants (*Elephas maximus*) display elevated fGCM concentrations in response to physical injury. To our knowledge, no data exist on the effect of physical injury on adrenal activity for new or old-world primates; therefore, this may be the first, albeit brief, look into this topic.

Similar to the male in the study, the female exhibited a prolonged elevation of fGCM concentrations throughout the post-injection period, presumably because of the limited visual, olfactory contact with the male following ACTH injection. The inability of the female to interact with the male may explain why the elevated fGCM concentrations did not return to baseline levels. Social instability and pair separation has been shown to increase the stress response and GC production in female squirrel monkeys (*Saimiri sciureus*, Lyons, Ha & Levine 1995) as well as common marmoset (*Callithrix geoffroyi*, Smith, Birnie & French 2011). The disruption of social bonds, through the separation of individuals, is known to evoke a significant bio-behavioural stress response, while the maintenance of established pair bonds can act as a ‘social buffer’ to both intrinsic and extrinsic stressors (Hennessy 1997). Thus, social buffering may assist in moderating the HPA response, decreasing GC production and stress-related behaviour (Hennessy, Kaiser & Sachser 2009). However, it is worth noting that the physiological response to social stressors can vary significantly between individuals and species; in this regard Foerster, Cords and Monfort (2011) demonstrated that individual rates of agonism in *C. mitis* showed no measureable fGCM response.

It should be noted that the prolonged elevation of fGCM concentrations from the initial increase post-injection, following the biological stressors perceived in both study animals, aggravates the interpretation of gut passage time within the study animals and additional research will have to be conducted to confirm the length found here.

A clear difference in signal strength to the biological and physiological stressors was observed when comparing the respective results from the different EIAs between sexes. The most prominent of which was the lack of a response observed in the female, at 23 h post-injection, compared to the male when using the cortisol EIA. Similarly, all three EIAs demonstrated a drastic adrenal response, in terms of fGCM secretion, during the biological stressor perceived by the female, whereas the respective response was considerably lower across all three EIAs in the male. In trying to explain these sex-related differences, a potential sex-related difference in GC metabolism and excretion should be considered (see review: Goymann 2012). For example, Touma et al. (2003) demonstrated that corticosterone metabolism in mice (*Mus musculus*) differ substantially between male and female individuals. Furthermore, the perception of a stressor can vary significantly between individuals and sexes (Goymann 2012; Koolhaas et al. 2010; see Stroud, Salovey & Epel 2002). For example, Suomi (1991) showed that the activation of the stress response, and the degree of GC secretion, differs substantially between individual rhesus monkeys (*Macaca mulatta*) faced with a similar social context. Another factor that may explain the observed difference in response between sexes is the nature of the biological stressor experienced by each sex. The instability in social structure, such as the separation of individuals from conspecifics, has been shown to be a major stressor in female individuals and may explain the considerable fGCM increase seen in this regard (Haller et al. 1999). With regard to the male, pain and discomfort have been shown to activate the stress response as discussed previously; however, the nature of the injury and the medical attention received by the male individual may have resulted in a lower stress response than that observed in the separation event of the female. Thus, the differences observed between the sexes may well be explained by the stressor type and not as a result of the specific steroid metabolism of each sex. To pinpoint respective responses, additional research into the specific factors that control stress physiology of each sex would be required.

From a technical perspective, both the 11-oxoetiocholanolone I and 11-oxoetiocholanolone II EIA were able to reliably monitor male and female alterations in fGCM concentrations across both the physiological and biological stressor periods; thus, both EIAs seem suitable to monitor adrenocortical activity in *C. albogularis erythrarchus*.

## Conclusion

Two of the three assays used in the study were able to monitor changes in fGCM concentrations of *C. albogularis erythrarchus* male and female study animals. Taking the MAD values and percentage response into consideration, the 11-oxoetiocholanolone I EIA was chosen as the most appropriate EIA for monitoring physiological stress in the species. The non-invasive technique validated for *C. albogularis erythrarchus* now offers conservationists, managers and academic researchers a robust and feedback-free tool for monitoring the physiological stress experience in both captive and free-ranging environments.
